# Exploring the Multifunctional Benefits of Astaxanthin in Aging, Oxidative Stress, Immune Dysfunction, Gut and Skin Health

**DOI:** 10.3390/antiox15050575

**Published:** 2026-05-02

**Authors:** Abdallah A. Basher, Nasir A. Ibrahim, Hao-Yu Liu, Nosiba S. Basher, Mohamed Osman Abdalrahem Essa, Hosameldeen Mohamed Husien, Saber Y. Adam, Demin Cai

**Affiliations:** 1 Jiangsu Key Laboratory of Animal Genetic Breeding and Molecular Design, College of Animal Science and Technology, Yangzhou University, Yangzhou 225009, China; mh24012@stu.yzu.edu.cn (A.A.B.); 007725@yzu.edu.cn (H.-Y.L.); 008643@yzu.edu.cn (H.M.H.); 2 Department of Biology, College of Science, Imam Mohammad Ibn Saud Islamic University (IMSIU), Riyadh 11623, Saudi Arabia; naabdalneim@imamueud.sa (N.A.I.); nsbasher@imamu.edu.sa (N.S.B.); 3 International Joint Research Laboratory, Universities of Jiangsu Province of China for Domestic Animal Germplasm Resources and Genetic Improvement, Yangzhou 225009, China; 4 College of Veterinary Medicine, Yangzhou University, Yangzhou 225009, China; dh23054@stu.yzu.edu.cn; 5 Biomedical Research Institute, Darfur University College, South Darfur State, Nyala 63313, Sudan

**Keywords:** natural compounds, antioxidant, anti-aging, Nrf2, inflammation, neuroprotection, carotenoids

## Abstract

Astaxanthin (AST) is a potent carotenoid renowned for its exceptional antioxidant properties, which has attracted considerable scientific interest due to its broad spectrum of health benefits. This review comprehensively evaluates the therapeutic potential of AST in counteracting age-related decline, oxidative stress, and immune dysfunction, while also examining its beneficial effects on gut and skin health. Current evidence demonstrates that AST effectively mitigates oxidative stress and supports cellular health and longevity by neutralizing free radicals and upregulating endogenous antioxidant systems. In addition, AST modulates immune responses under conditions of immune dysfunction, thereby enhancing resilience against inflammatory disorders and infections. Emerging studies further indicate that AST promotes gut health by improving intestinal barrier integrity and maintaining a balanced gut microbiota, both of which are essential for systemic well-being. Moreover, its capacity to enhance skin elasticity and protect against ultraviolet-induced damage underscores its promising applications in cosmetic and dermatological products. This review highlights the urgent need for additional well-designed clinical trials to fully elucidate the underlying mechanisms, optimal bioavailability, dosage regimens, and long-term safety of AST. By integrating findings across multiple research domains, the present work provides a concise yet comprehensive overview of AST as a promising nutraceutical for promoting health, healthy aging, and the management of chronic diseases.

## 1. Introduction

**Figure 1:**
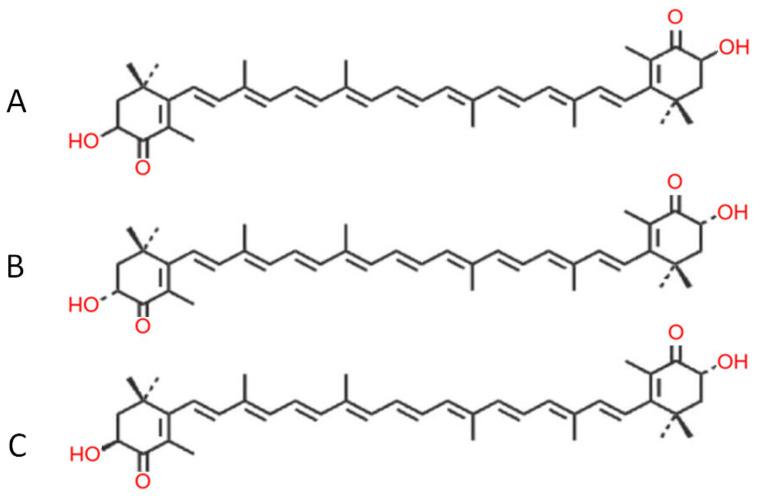
Chemical structures of AST: (**A**) 3S,3′S-AST; (**B**) 3R,3′S-AST; (**C**) 3R,3′R-AST.

Astaxanthin (AST) is a lipid-soluble, red-orange oxycarotenoid pigment [1]. It serves as a key colorant in aquaculture feeds for salmonids and crustaceans. As a xanthophyll carotenoid, AST belongs to the broader carotenoid family, which also comprises lutein, canthaxanthin, β-cryptoxanthin, and zeaxanthin [2]. Its natural sources are diverse and include yeasts, algae, and various marine animals (Table 1) [3]. Commercially, AST is primarily obtained through extraction from the microorganisms *Blakeslea trispora*, *Phaffia rhodozyma*, and *Haematococcus pluvialis*, or via chemical synthesis [4]. The green *microalga H*. *pluvialis* accumulates AST as a protective response to environmental stressors, including high irradiation, nutrient starvation, elevated salinity, intense light, and extreme temperatures [5]. Consequently, AST is present in krill, shrimp, salmon, crayfish, and trout, which acquire the pigment by consuming AST-rich algae or other marine organisms [6]. Although humans cannot synthesize AST endogenously, it is readily obtained through the diet, particularly from seafood [7]. Naturally occurring AST exists in multiple forms, including stereoisomers, geometric isomers, and both free and esterified configurations. Chemically, AST (3,3′-dihydroxy-4,4′-diketo-β,β′-carotene; molecular formula C_40_H_52_O_4_; molar mass 596.84 g/mol) consists of 40 carbon atoms arranged as two oxygenated β-ionone rings connected by a central polyene chain [8,9,10]. Because of its high susceptibility to oxidation and inherent chemical instability, AST in nature is predominantly found esterified with one or two fatty acids (forming mono- or di-esters) or bound to proteins, as observed in lobster exoskeletons and salmon muscle [11]. Synthetic AST, in contrast, may occur in chiral ((3S,3′S) or (3R,3′R)) or meso (3R,3′S) forms (Figure 1), with the chiral stereoisomers being the most common. Additionally, the polyene chain can adopt trans or cis configurations; however, owing to the thermodynamic instability of the cis isomers, the trans configuration predominates in both natural and synthetic AST [12].

### 1.1. Astaxanthin Regulatory Landscape

The regulatory landscape for AST differs internationally and is largely contingent upon its origin (natural vs. synthetic) and purpose of use. In the United States, the regulation of supplements and food additives is managed by frameworks such as the Dietary Supplement Health and Education Act (DSHEA) and the Federal Food, Drug, and Cosmetic Act. Natural AST sourced from *Haematococcus pluvialis* has been granted Generally Recognized As Safe (GRAS) status by the U.S. Food and Drug Administration (FDA) for designated uses in foods and dietary supplements. The permissible levels of AST in food supplements were up to 8 mg per day, and the acceptable daily consumption for adults varied from 0.034 to 0.2 mg AST/kg body weight [13]. This classification indicates that certified professionals consider it safe for its intended usage conditions. Microalgae-derived AST serves as a pigment in aquaculture feeds and functions as a nutraceutical [14]. Synthetic AST, although extensively utilized in the aquaculture sector for its economic advantages, encounters heightened regulatory oversight about human intake [15,16]. It is widely recognized as a feed supplement for animals, especially for enhancing pigmentation in aquaculture species such as salmon, trout, and crustaceans [17]. Nonetheless, its endorsement for direct human supplementation necessitates individual assessment and lacks the universal acknowledgment afforded to natural AST.

### 1.2. Safety of Astaxanthin

The long-term safety profile and regulatory status of AST significantly differ between its natural and synthetic variants. Natural AST, especially derived from *Haematococcus pluvialis*, has a longstanding record of safe human consumption exceeding two decades, and is classified as GRAS in the United States, along with receiving approval from the European Food Safety Authority (EFSA) [18]. Synthetic AST, in contrast, lacks equivalent long-term safety data for humans and is typically not authorized for dietary supplements in significant jurisdictions. Its principal application is confined to animal feed, specifically in aquaculture, where it is utilized to augment the pigmentation of cultivated aquatic species such as salmon, shrimp, and rainbow trout [15,19]. The rigorous regulatory oversight for compounds purporting to treat diseases or those substantially modified from their natural form highlights the significance of a “do not harm” approach. The process of introducing a medicine or supplement to the market necessitates comprehensive testing for identification, purity, potency, and safety, hence ensuring product consistency and reliability. Synthetic technologies provide economical production; nevertheless, apprehensions about possible impurities or hazardous by-products restrict their applicability in human applications [20].

A number of studies and experiments have demonstrated that AST has a good safety record. Safety testing of an AST-enriched extract at 6 or 20 mg daily for 8 weeks and 4 weeks, respectively, in healthy adults revealed no changes in blood chemical, haematological, or blood pressure markers that were clinically important [21]. Likewise, taking 8 mg of AST daily for three months did not result in any gastrointestinal distress or any other adverse effects [22,23]. Patients with age-related macular degeneration who received a daily oral supplement containing 4 mg of AST for a period of 12 months did not notice any adverse reactions or toxicity [24], nor in those with practical dyspepsia who received *H. pluvialis* AST at 40 mg daily for 4 weeks [25], neither at a dose of 20 mg/day during three weeks in obese adults and overweight [26]. Similarly, in healthy men, there were no detrimental side effects after a single administration of 100 mg AST orally equivalents from its fatty acyl diesters [27] or free AST [28]. For example, rats given large doses (up to 1240 mg/kg/day) orally over a lengthy period of time (90 days) showed no harmful effects [29]. Moreover, the 13-week repeated oral administration of a natural AST-rich extract (ARE) from *P. carotinifaciens* to rats at dosage levels of 250, 500, and 1000 mg/kg/day did not result in any significant toxicological alterations [30]. Even when compared to the placebo in randomized trials, AST exhibits excellent clinical safety at low (up to 12 mg) or high (up to 100 mg) daily dosages [31]. No significant toxicities have been identified for this organism; consequently, the dry cell biomass has received approval from the US FDA and the European Food Safety Authority (EFSA) for use as a natural colouring agent in salmon and trout aquaculture [32]. For the past decade, this whole cell, AST-rich substance, commercially known as Panaferd^®^, has been widely utilised in the production of organic salmon and trout for human consumption. The findings suggest that AST supplementation at the specified dosage level is generally safe and well tolerated.

### 1.3. Astaxanthin Bioavailability

The bioavailability of AST is a crucial determinant of its effectiveness. Natural AST, especially the esterified forms present in *Haematococcus pluvialis*, typically has superior bioavailability relative to synthetically produced AST [33,34]. The enhanced bioavailability is due to better micellarization and lymphatic absorption of the esterified natural form. Research has demonstrated that natural sources can result in plasma Area Under the Curve (AUC) values in people that are two to three times greater than those of synthetic forms. The limited oral bioavailability of AST, a highly lipophilic molecule, poses a considerable impediment, restricting its extensive use in food and nutritional sectors [33,35,36]. To address this issue, multiple ways are being investigated to improve the bioavailability of AST, such as encapsulating techniques and emulsion systems [36,37]. Stable oil-in-water emulsions stabilized by casein–caffeic acid–glucose ternary conjugates exhibit enhanced resilience to unfavorable gastrointestinal conditions, potentially augmenting AST administration [36]. The chemical structure, transport proteins, food matrix effects, and gut flora all influence the oral bioavailability of AST, demanding further research to enhance its absorption. The debate between natural and synthetic AST is multifaceted, encompassing efficacy, safety, and market preference (Table S1).

AST prevents oxidative damage at the cellular and molecular levels by scavenging radicals, preventing lipid peroxidation, controlling OS-related gene expression, and quenching singlet oxygen [38]. AST enhances immune response and inflammation management and is a biomarker for oxidative damage to deoxyribonucleic acid (DNA) in young, healthy females. However, in circumstances of extreme oxidative stress and in immune compromised patients, antioxidants typically exhibit more significant physiologic regulation [39]. Although it is not meant to be comprehensive, the goal of this chapter is to summarize the well-researched activities and their likely modes of action.

**Table 1:** Sources of AST from natural and genetically modified organisms.

Group of Organisms	Representative	References
Animalia	*Redfish Crustaceans* (*Pandalus borealis*, *Euphausia superba*, *Calanus finmarchicus*, etc.) *Wild salmon* (*Oncorhynchus species*)	[40,41]
Plantae (microalgae)	*Chlorella zofingiensis*, *Chromochloris zofingiensis*, *Chlorococcum*, *Chlamydomonas reinhardtii*, *Diatoms*	[42,43]
Lichen	*Clodia aggregata*, *Concamerella fistulata*, *Usnea amaliae*, *Usnea densirostra*	[44]
Fungus (yeasts)	*Xanthophyllomyces dendrorhous* (*Phaffia rhodozyma*), *Yarrowia lipolytica* ^®^, *Saccharomyces cerevisiae* ^®^	[45,46]
Crustaceans	*Haematococcus pluvialis*	[47,48]
Bacteria	Cyanobacteria (*Synechococcus* sp.), *Corynebacterium glutamicum* ^®^, *Paracoccus carotinifaciens*, *Agrobacterium aurantiacum*, *Escherichia coli* ^®^	[49,50]

Notice; ^®^ genetically modified organism (GMO).

## 2. Literature Search Strategy

A systematic literature search was conducted to ensure a comprehensive, transparent, and reproducible synthesis of the existing information on the multifunctional advantages of AST. A methodical search technique was implemented in this study to reduce selection bias and capture the extensive literature on AST’s impact on aging, oxidative stress, immunological dysfunction, gut health, and skin conditions.

### 2.1. Search Databases, Sources, Strategy, and Keywords

A comprehensive literature search was conducted utilizing two primary electronic databases: PubMed/MEDLINE, and Scopus. These databases were chosen for their comprehensive coverage of biological, nutritional, and pharmaceutical sciences. Furthermore, the reference lists of qualifying full-text articles and relevant review papers were manually examined (snowballing) to uncover any further studies not included in the initial electronic search.

The search strategy was designed utilizing a blend of restricted vocabulary (MeSH terms in PubMed). The principal search phrases were organized around the fundamental elements of the review: the compound, its biological effects, and the specific health domains addressed. Boolean operators (AND, OR) were employed to amalgamate terms. A composite search string employed was (“Astaxanthin”[MeSH] OR “astaxanthin”[Title/Abstract]) AND (“Oxidative Stress”[MeSH] OR “aging”[Title/Abstract] OR “Immune System”[MeSH] OR “Gastrointestinal Microbiome”[MeSH] OR “Skin”[MeSH]).

### 2.2. Inclusion and Exclusion Criteria

The review focused on the multifunctional advantages relevant to human health and the underlying mechanisms, utilizing the following criteria: Inclusion Criteria: Peer-reviewed original research articles (including in vitro, in vivo, and clinical investigations) and review articles published in English. Research examining the impact of AST (from both natural and synthetic origins) on aging biomarkers, oxidative stress mechanisms, immunological modulation, gut microbiota composition, intestinal barrier integrity, and skin condition. Articles published between 1990 and 2025 to capture the modern era of nutraceutical research while including seminal older studies. Criteria for Exclusion: Conference abstracts, editorials, opinion articles, and items in languages other than English. Research solely focused on aquaculture or the utilization of AST as a feed supplement, devoid of translational relevance for human health. Research involving AST alongside other chemicals, where the unique effect of AST could not be discerned. The gathered data from these papers encompassed the author, year of publication, study design, model system, dosage of AST, length of intervention, and principal findings relevant to the themes of aging, oxidative stress, immunity, gut health, and skin health. This systematic method of literature collection ensures that the narrative in this review is anchored in a robust and well-defined evidence basis.

### 2.3. Study Selection and Data Extraction

**Figure 2:**
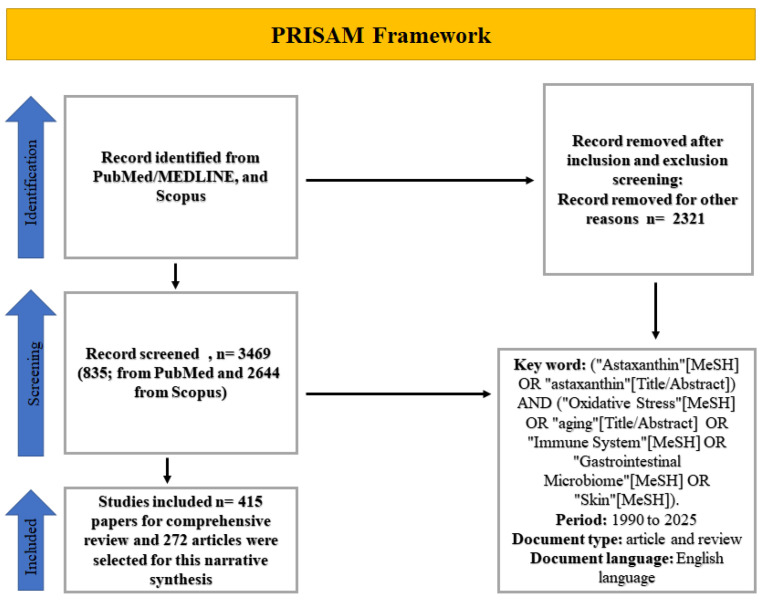
PRISMA framework for the review of AST in aging, oxidative stress, immune dysfunction, gut, and skin health.

The preliminary search conducted on 2 January 2026, resulted in 3469 articles from the two databases (PubMed: 835; Scopus: 2644). Following the elimination of duplicates via reference management software (EndNote, version 21), 876 articles persisted. These were evaluated based on title and abstract, yielding 415 papers for comprehensive review. After applying the inclusion/exclusion criteria, 272 articles were selected for this narrative synthesis, (Figure 2). The data obtained from these papers comprised the author, year of publication, study design, model system (e.g., human, rodent, cell line), dosage of astaxanthin, length of intervention, and principal findings pertinent to the themes of aging, oxidative stress, immunity, gut health, and skin health.

## 3. Astaxanthin and Aging

Aging is naturally accompanied by elevated free radical production and declining cellular energy metabolism. Biologically, the aging process is driven by the progressive accumulation of oxidative damage within cells and tissues [3]. As individuals advance in age, they exhibit a broad spectrum of health issues that vary widely among people, depending on lifestyle, genetic background, environmental exposures, and life experiences [51]. Adherence to a healthy lifestyle combined with a balanced, nutrient-dense diet is strongly associated with successful aging and prolonged periods of optimal health [52]. The two primary strategies for extending healthy lifespan are pharmacological (or genetic) interventions and lifestyle modifications. According to Liu J.K. [53] biogerontological research offers considerable potential for the pharmaceutical and healthcare sectors, as anti-aging therapies target enhanced cellular regeneration, autophagy induction, epigenetic modulation of gene expression, and caloric restriction. This field employs state-of-the-art scientific and medical technologies to prevent, diagnose, treat, and reverse age-related functional decline. Crucially, the main objective of anti-aging interventions is not merely to increase lifespan but to sustain health and quality of life for an extended duration [52]. Rattan has therefore proposed shifting the focus of the field from “anti-aging” to “healthy aging” to prioritize research centered on health promotion [54]. This reorientation largely explains why specialists now predominantly use the term “healthy aging” rather than “anti-aging” [52]. In addition, natural products can improve overall health, extend lifespan, and enhance well-being by attenuating the development of several age-related chronic conditions, including cardiovascular disease, diabetes, cancer, and neurodegeneration.

AST represents 54a highly promising candidate geroprotector among the various naturally derived molecules. Although the exact mechanisms by which AST promotes these effects have yet to be determined, several studies have shown that it can stimulate a variety of biological mechanisms at the cellular level, including the modulation of transcription factors and genes that are directly linked to longevity processes. One of the primary evolutionarily conserved transcription factors controlled by AST is the forkhead box O3 (FOXO3) gene, which has been recognised as a key modulator of brain cell destiny and function [55,56]. Furthermore, It effectively shields the double membrane system of the mitochondria, improving its capacity to generate energy [57]. Study in vitro has stated that AST can increase the growth of precursor cells of brain. In vitro studies suggest that AST may actually improve the ability of neural progenitor cells (NPCs) to divide and form colonies in a time and dose-dependent manner by strongly activating phosphoinositide 3-kinase (PI3K) and its downstream mediators. In addition to acquiring active self-renewal activity by overexpressing canonical stemness genes like octamer-binding transcription factor 4 (OCT4), SOX2, Nanog, and Kruppel-like factor 4 (KLF4), it upregulated important transcription factors associated with proliferation, including Rex1, cyclin-dependent kinase 1 (CDK1), and 2 [58]. These in vitro findings on cell proliferation were recently confirmed on mice (11 weeks) fed a diet supplemented with AST for 4 weeks. Thus, as demonstrated by the enhanced immunohistochemical staining of bromodeoxyuridine (BrdU), the AST-enriched diet was able to promote proliferation of the cell in the dentate gyrus (DG), improving hippocampal-dependent cognitive abilities [59]. AST benefited older, healthy people’s cognitive function. AST may help prevent age-related cognitive decline, according to a human experiment (*n* = 44) that evaluated AST supplementation with a 12 mg daily dose for 12 weeks [60]. Furthermore, to assess the impact of AST on aging, a randomized clinical experiment was conducted with 32 healthy individuals aged 60–70 who had verified symptoms of oxidative stress. This study showed that administering of co-crystallized AST at 7 mg with enhanced bioa60vailability to a lysosomal dark chocolate formulation has interesting effects on improving oxidative state in elderly human subjects. It advised the potential benefits of a combination of dark chocolate with AST [61]. AST impact on brain plasticity and cognitive performance was evaluated in a recent study using both young and old mice. A month of AST supplementation improved hippocampal cognitive function and promoted long-term potentiation (LTP) in aged mice, offering compelling proof that AST supplementation can result in visible cognitive enhancements along with beneficial behavioural outcomes [62]. Supplementing with AST may influence neurogenesis and synaptic plasticity by producing an elevation of Brain-Derived Neurotrophic Factor (BDNF), suggesting the possibility of an efficient activator [63]. Furthermore, another interesting target of AST-mediated neuroprotection is the evolutionarily conserved forkhead box O (FOXO) family of transcription factors. One possible way that AST may impact adult neurogenesis is by activating FOXO3, a transcription factor that may have a direct impact on the genetic network that maintains a healthy pool of mammalian stem cells for lifelong neurogenesis [56]. Having been considered, AST is a promising nutraceutical supplement for treating a range of diseases, including hair loss, where oxidative stress and inflammation are key factors in the development and subsequent progression of the condition. AST also alters a number of significant cell-signaling pathways, such as the JAK-STAT, PPARγ, and NF-kB pathways [3].

Inflammation, commonly termed “inflammaging” in relation to aging, plays a substantial role in age-associated diseases. Senescent cells aggregate in tissues with aging and exude a pro-inflammatory mixture termed the senescence-associated secretory phenotype (SASP), which can harm adjacent tissues and sustain a chronic inflammatory condition [64]. The anti-inflammatory characteristics of AST, partially mediated by the inhibition of the NF-κB pathway, help mitigating systemic inflammation [65]. This is especially pertinent in diseases such as vascular calcification, where oxidative stress and inflammation are involved, and AST has shown potential in mitigating this process in vitro [66]. Moreover, the pathways via which AST demonstrates its anti-aging properties are varied and encompass more than simply antioxidant activity. The Nuclear factor erythroid 2-related factor 2 (Nrf2)/Keap1 pathway is a crucial route regulated by AST. Nrf2 serves as a principal regulator of intrinsic antioxidant and detoxifying enzymes. The activation of Nrf2 by AST results in the overexpression of protective enzymes, hence augmenting cellular defense against oxidative stress [65,67]. Research indicates that AST mitigates oxidative stress and immunological dysfunction in D-galactose-induced aging in rats by activating the Nrf2/Keap1 pathway and inhibiting the NF-κB pathway, which is implicated in inflammatory responses [65]. In addition, mitochondrial dysfunction is another hallmark of aging, characterized by impaired energy production and increased generation of reactive oxygen species (ROS) [68]. AST’s lipophilic characteristics facilitate its seamless incorporation into mitochondrial membranes, allowing it to immediately neutralize ROS and safeguard mitochondrial integrity [69]. Research demonstrates that AST can inhibit H2O2-induced excessive mitophagy and apoptosis in SH-SY5Y cells through the modulation of Akt/mTOR activation, consequently safeguarding mitochondrial function and enhancing cell viability [70]. The generation of superoxide and hydrogen peroxide in the mitochondrial matrix is primarily governed by site IQ of complex I, rendering mitochondrial protection a vital component of anti-aging treatments [71].

Expanding on the comprehension of AST impact on ageing, it is crucial to investigate the fundamental biological pathways that lead to age-related deterioration. Oxidative stress significantly contributes to the acceleration of cellular damage and functional decline. Investigating the correlation between AST and oxidative stress yields significant insights into its potential as a natural antioxidant, emphasising how this carotenoid may alleviate oxidative damage and promote healthy ageing at the molecular level. Table 2 lists some clinical studies investigating the possible use of AST in the treatment of aging-related diseases.

## 4. Astaxanthin and Oxidative Stress

Oxidative stress arises from an imbalance between the production of oxidants and the capacity of the cellular antioxidant defense systems, resulting in the excessive accumulation of reactive oxygen species (ROS) and free radicals. These highly reactive molecules can interact with lipids, proteins, and DNA, triggering chain reactions that lead to lipid peroxidation, DNA damage, and protein dysfunction, ultimately contributing to the pathogenesis of various diseases [80,81]. Oxidative stress is widely recognized as a central driver of inflammation, cancer, autoimmune disorders, cardiovascular diseases, neurodegenerative conditions, arthritis, and aging itself [82,83]. In addition, it induces epigenetic alterations, including global DNA hypomethylation that promotes genomic instability and proto-oncogene activation, as well as localized hypermethylation of tumor-suppressor gene promoters that results in gene silencing [84]. Consequently, mitigating the detrimental effects of oxidative stress through diverse interventional strategies is essential for preserving health and ensuring a high quality of life.

AST exhibits superior antioxidant activity compared with other carotenoids, including lutein, α-carotene, β-carotene, and lycopene [85]. For instance, AST is approximately 550 times more effective than α-tocopherol at neutralizing singlet oxygen (^1^O_2_) [86]. This exceptional potency originates from the combination of the conjugated polyene chain common to all carotenoids and the unique terminal rings that are characteristic of the AST structure [87]. This design allows AST to cross the whole lipid bilayer, attaching to both the inner and outer membrane surfaces. Carotenoids without polar end groups, such as β-carotene and lycopene, cannot interact with aqueous-phase radicals or stabilise membrane interfaces, they are located deeper within the hydrophobic core. This trans membrane orientation enables AST to intercept free radicals at many locations and block the propagation of lipid peroxidation chain reactions more effectively than its equivalents [88]. Studies indicate that natural AST is a rich component for formulations promoting healthy aging and a very effective ROS scavenger. It decreased oxidative stress in patients and enhanced the serum lipid profile via the normalization of serum triglycerides and the elevation of good HDL-cholesterol [89]. Furthermore, AST enhances mitochondrial membrane integrity and protects mitochondria from oxidative damage, resulting in improved cellular energy status and greater ATP-generating capacity [90]. Nuclear factor erythroid 2-related factor 2 (Nrf2) is a master transcription factor that maintains redox homeostasis and represents a promising therapeutic target for conditions associated with inflammation and oxidative stress [91,92]. Upon activation, Nrf2 induces the expression of numerous cytoprotective and antioxidant genes, including heme oxygenase-1 (HO-1), glutathione S-transferase 1 (GST-1), and NADPH quinone dehydrogenase 1 (NQO1) [93]. AST has been reported to activate and upregulate the Nrf2 pathway by stimulating the PI3K/Akt and ERK signaling cascades [94]. Although the precise effects of AST on Kelch-like ECH-associated protein 1 (Keap1) remain unclear owing to inconsistent findings across studies [95]. Evidence suggests that AST inhibits Nrf2 degradation and thereby disrupts its interaction with Keap1 [96]. Although AST can increase ERK activity, it does not require ERK activation to disrupt the Keap1/Nrf2 complex, suggesting that AST increases Nrf2 nuclear translocation through a different mechanism [97]. For example, in TPhP-induced neurodevelopmental toxicity, AST stimulated the Nrf2/Keap1/HO-1 pathway to reduce oxidative stress and ferroptosis [97]. AST upregulates enzymes such as superoxide dismutase (SOD) and glutathione peroxidase (GPx), which aids in the efficient neutralization of ROS. SOD transforms superoxide radicals to hydrogen peroxide, which is then detoxified by CAT and GPx into water [98]. For example, AST supplementation in head and neck cancer patients receiving cisplatin chemotherapy has been demonstrated to raise SOD levels and decrease malondialdehyde (MDA) levels [98]. This coordinated enzymatic action greatly decreases ROS levels and prevents cellular damage.

After 12 randomized clinical trials with 380 people, Ma and colleagues discovered that AST might lower oxidative stress and inflammation biomarker levels. In overweight patients, its use decreased serum isoprostane content, enhanced SOD activity, and decreased blood MDA concentration [26,39,99,100,101,102]. Similarly, AST reduced hippocampal damage caused by lanthanum oxide nanoparticles by lowering oxidative stress and neuroinflammation through the PI3K/Akt/Nrf2 pathway [103]. According to a recent study in vitro, adding 20 µM ASX to MCF-7 cells increased ROS by 53.3% compared to 17.3% for the control, indicating significant pro-oxidant activity. Based on the study’s findings, this effect improved the synergistic treatment of cells with a combination of lutein, β-carotene, and AST (68.1% increase in ROS) [104]. This pro-oxidant action is not a general AST characteristic, but rather an exploitable vulnerability in cancer cells, those results from their particular metabolic architecture. Cancer cells, including MCF-7, frequently exist in a state of elevated basal oxidative stress because of their high metabolic rate, oncogenic signalling, and mitochondrial dysfunction [105]. To survive this self-imposed stress, they upregulate their antioxidant defence mechanisms, creating a more delicate redox balance than that of normal cells. In addition, AST may prevent doxorubicin-induced memory impairment and lessen neuroinflammation, oxidative stress, and impairment of the memory caused by lipopolysaccharide (LPS) via preventing inflammatory, oxidative, and pro-apoptotic insults [106,107]. These results indicate that despite AST’s well-known antioxidant qualities, which protect cells from oxidative damage, it may cause oxidative stress in cancer cells [39,108].

AST’s protective effects also include the modulation of other critical signaling pathways that control cellular metabolism, stress responses, and longevity. The Sirtuin 1 (SIRT1)/AMPK/FOXO1 pathway is very important in diseases such as diabetic retinopathy. SIRT1, a NAD+-dependent deacetylase, plays an important function in preventing mitochondrial damage and apoptosis [109]. AMP-activated protein kinase (AMPK) is an important energy sensor that, when active, promotes catabolic processes that produce ATP while inhibiting anabolic processes that use ATP. AST protects retinal Müller cells from high glucose-induced oxidative stress by activating the SIRT1/AMPK/FOXO1 pathway [109]. This activation contributes to the reduction in mitochondrial damage and apoptosis, both of which are important events in DR pathogenesis. Similarly, in mouse preantral follicles, AST promoted follicular growth through the AMPK signaling pathway, indicating a more general involvement in metabolic regulation and cellular resilience [110]. The interaction between AST and these pathways emphasizes its diverse impact on cellular homeostasis and its potential therapeutic uses in metabolic illnesses and age-related disorders. Hence, further clinical study is needed for more clarification regarding the mechanisms of AST during oxidative stress. Oxidative stress is intricately linked to the dysregulation of immune responses, contributing to immune dysfunction and increased susceptibility to various diseases. Therefore, investigative the role of AST in modulating immune function not only extends our comprehension of its therapeutic potential but also highlights its significance in maintaining overall health and resilience against immune-related disorder.

## 5. Astaxanthin and Immune Dysfunction

Immunodeficiency is a condition where the capacity of the immune system to combat pathogens, such as viruses or bacteria, is weakened. This happens when the immune system is not functioning at all or operates inefficiently [111]. Contributing factors include an unhealthy lifestyle, obesity, malnutrition, smoking, excessive alcohol consumption, certain diseases, and genetic predispositions. In addition, unresolved inflammation can exacerbate various chronic conditions, including rheumatoid arthritis, atherosclerosis, inflammatory bowel disease, neurological disorders, and chronic obstructive pulmonary disease [112,113]. A wide array of inflammatory biomarkers have been identified, including acute-phase proteins (APPs), cytokines, chemokines, immune-related effectors, reactive oxygen and nitrogen species (RONS), prostaglandins, platelet-activating factor (PAF), cyclooxygenase-related factors, growth factors, transcription factors, and key signaling pathways such as MAPK, NF-κB, and JAK-STAT [114].

AST exerts significant protective effects against immune dysfunction, particularly in the presence of inflammation. Its mechanisms of action include the modulation of inflammatory cytokines, enhancement of antioxidant defenses, and regulation of immune cell functions. AST has been shown to inhibit the induction and expression of iNOS and COX-2 proteins, thereby suppressing the production of inflammatory mediators in LPS-stimulated BV-2 microglial cells [115]. In a study by Park et al. [116] AST was found to attenuate inflammation in streptozotocin (STZ)-induced diabetic mice by downregulating the expression of COX-2, iNOS, and ICAM-1 proteins. Additional studies have confirmed that AST suppresses inflammation induced by excessive physical exercise [102] and ultraviolet (UV) irradiation, through the downregulation of iNOS and COX-2 at both the protein and mRNA levels [117]. Several reports have further indicated that AST reduces inflammatory responses by decreasing the gene expression of key mediators, such as tumor necrosis factor and interleukin-8. Moreover, AST has been shown to inhibit NF-κB kinase activation in human keratinocytes, offering a novel therapeutic approach for the management of inflammatory skin disorders [118]. Numerous in vivo and in vitro studies have demonstrated that AST modulates immune responses. In vitro experiments have revealed that, under T cell-dependent stimulation, AST enhances immunoglobulin production in human lymphocytes, particularly T-helper cells [119]. AST has also been found to activate NK cells and T cells, which play critical roles in immune regulation and B-cell differentiation [39]. In addition, AST exhibits potential anti-tumor properties [120], At low-to-moderate doses (typically 1–20 mg/kg in murine models), AST consistently improves multiple aspects of anti-tumor immunity. This optimal dose range significantly enhances NK cell cytotoxicity, increases CD8+ T-cell infiltration into tumour microenvironments, and promotes dendritic cell maturation [121,122]. The mechanism driving this enhancement entails AST’s selective accumulation in lymphoid tissues and its capacity to differentially modulate redox-sensitive signalling pathways among immune cell types. Specifically, AST activates the Nrf2/Keap1 antioxidant pathway while concurrently inhibiting NF-κB-mediated pro-inflammatory signalling, fostering an environment conducive to adaptive immune function without inciting excessive inflammation [123]. Studies indicate that AST plays a part in improving immune function overall by increasing natural killer (NK) cell activity and proliferation of the lymphocyte [124]. In LPS-challenged mice, oral AST administration significantly increased survival rates, suggesting that it may have therapeutic use in sepsis [125]. On the other hand, although AST exhibits promise in regulating immune responses, the complexity of immune dysfunction suggests a multimodal strategy could be required for successful therapy, since other factors can also affect immune health. In addition to its immunomodulatory effects, AST’s impact on the gastrointestinal system, which is crucial for general health, must also be considered. The gut is essential for nutrient absorption and functions as a vital element of the immune system, providing a barrier against infections and influencing immunological responses. A comprehensive understanding of the interactions between AST and gut health provides deeper insights into its potential as a versatile agent for promoting systemic well-being. Table 3 summarizes selected protective effects of AST mediated through the modulation of inflammatory pathways.

## 6. Astaxanthin and Gut Health

The stomach and intestines represent the primary digestive organs responsible for the chemical and physical breakdown of food and the subsequent absorption of nutrients [161]. The gastrointestinal (GI) mucosa is constantly exposed to diverse oxidative stressors and inflammatory stimuli, including cigarette smoking, chronic alcohol consumption, *Helicobacter pylori* (*H. pylori*) infection, and nonsteroidal anti-inflammatory drugs (NSAIDs) [162,163]. The complexity of gut diseases is highlighted by the fact that, although microbial and genetic variables are frequently the focus, personal choices and environmental exposures also have a major impact. Several studies have demonstrated that AST effectively modulates conditions associated with gut health.

### 6.1. Gastric Cancer

Gastric cancer remains one of the leading causes of cancer-related mortality worldwide, despite a notable decline in its incidence over the past decade [164]. Although the precise etiology of the disease has not been fully elucidated, multiple risk factors contribute to its development, including *H. pylori* infection and dietary habits such as the consumption of highly salted foods. Smoking further elevates the risk of gastric cancer, as nicotine strongly promotes metastasis by activating the ROS/MAPK (ERK1/2, p38)/AP-1 and ROS/NF-κB signaling axes in AGS gastric cancer cells. This process upregulates IL-8 expression and stimulates angiogenesis as well as endothelial cell proliferation [165]. In addition, a meta-analysis has demonstrated that alcohol consumption significantly increases the risk of gastric cancer [166]. Reactive oxygen species (ROS) play a central role by regulating p38 MAPK, which triggers cell death and activates multiple downstream signaling pathways—including p53, Wnt, Ras, and mTOR—thereby initiating gastric carcinogenesis [167]. Moreover, elevated ROS levels stimulate redox-sensitive transcription factors such as AP-1 and NF-κB, leading to enhanced expression of inflammatory genes and adhesion molecules that facilitate the invasion of gastric cancer cells [168].

AST treatment (1, 10, 50, or 100 µM for 48 h) significantly inhibited cell proliferation by arresting cell cycle progression in the human gastric cancer cell lines KATO-III and SNU-1. This anti-proliferative effect was mediated through the suppression of ERK phosphorylation and the upregulation of p27 expression, which in turn reduced the levels of key cell cycle regulators, including the cyclin D1/cyclin-dependent kinase 4 (CDK4) and cyclin E/CDK2 complexes, thereby inducing G0/G1 phase arrest [169]. Further transcriptomic array profiling in *H. pylori*-infected AGS cells demonstrated that AST treatment (5 µM for 3 h) markedly downregulated the expression of genes involved in cytoskeleton rearrangement, motility, and migration, such as c-MET, EGFR, PI3KC2, PLCγ1, Cdc42, and ROCK1 [170]. RNA sequencing (RNA-Seq) analysis of *H. pylori*-infected human gastric epithelial AGS cells revealed that AST (5 µM for 3 h) substantially suppressed the overexpression of several genes associated with the Wnt/β-catenin signaling pathway—including Fos-like 1, c-myc, and porcupine—which governs cell proliferation. In addition, AST effectively reversed the *H. pylori*-induced downregulation of the tumor suppressor genes Bambi and Smad4 in gastric epithelial cells. Furthermore, AST inhibited *H. pylori*-mediated Smox expression, thereby attenuating oxidative DNA damage [171]. In the study by Lee et al., pretreatment with AST (25 or 50 nM for 3 h) prevented *H. pylori*-induced apoptosis in AGS cells by blocking AMPK-mediated autophagy. Consequently, AST significantly reduced *H. pylori*-triggered cell death, caspase-3 activation, DNA fragmentation, and cytochrome c release. Mechanistically, AST activated AMPK, which inhibited mTOR signaling and thereby suppressed autophagosome formation [172].

### 6.2. Colon Cancer

Although the possibility of genetic predisposition, a number of epidemiological research have revealed that environmental factors, including heavy alcohol use, a diet low in fiber and rich in fat, and tobacco use, significantly influence the risk of colon cancer [173]. Despite the molecular processes by which ROS and inflammation contribute to carcinogenesis remain unclear, redox-sensitive transcription factors like AP-1, STAT3, and NF-κB have been firmly related to colon carcinogenesis [174]. Therefore, it has been discovered that a number of chemoprotective drugs, antioxidants, and anti-inflammatory bioactive chemicals offer molecular treatments for colon cancer by modifying aberrantly activated NF-κB, AP-1, and STAT3 [175,176].

AST has been demonstrated to control cancer hallmarks such as metastasis, apoptosis, and proliferation in a number of colon cancer models [122,177,178]. AST-rich *Haematococcus pluvialis* extract (25 µL/mL; 24 h) significantly increased the expression of p53, p21, and p27 in HCT-116 colon cancer cells while decreasing the expression of cyclin D1 and AKT phosphorylation. Furthermore, the *Haematococcus pluvialis* extract altered the Bax/Bcl-2 ratio and increased p38, JNK, and ERK1/2 phosphorylation [179]. These results suggest that AST may successfully suppress colon cell proliferation by preventing cell cycle progression and promoting apoptosis [179]. It is also possible that AST’s anti-cancer effects are mediated by microRNA (miR) regulation. By transcriptionally suppressing an oncogenic transcriptional factor, MYC, AST treatment (50–100 µM; 24 h) enhanced the production of miR-29a-3p and miR-200a, which are known to have an anti-metastatic effect, in colon cancer cell lines CT26 and HCT-116. Thus, elevated levels of miR-29a-3p and miR-200a resulted in reduced expression of MMP2 and zinc finger E-box binding homeobox 1 (ZEB1), which promote the epithelial–mesenchymal transition of colorectal cancer cells. The MYC/miR-29a-3p and miR-200a axis prevents colon cancer from spreading to the lung when AST therapy (25–50 mg/kg BW; four weeks) is administered to BALB/c nude mice that have been injected with colon cancer cells [180]. Furthermore, AST food supplementation (200 ppm; 17 weeks) alleviated colonic proliferative lesions in a mouse model of azoxymethane (AOM)/DSS-mediated colon carcinogenesis, possibly by inhibiting the production of NF-κB, TNF-α, and IL-1β in the malignancies pretreatment of AST (15 mg/kg BW; 16 weeks) significantly reduced the frequency of aberrant crypt foci and the severity of lesions in rats administered 1,2 dimethylhydrazine (DMH) to induce colon carcinogenesis [181]. In line with these findings, AST also reduced the incidence of colon cancer linked to obesity. In leptin receptor-null C57BL/KsJ-db/db mice injected with AOM, Kochi et al. showed that a diet containing AST (200 ppm; eight weeks) decreases oxidative stress by boosting the expression of SOD1, GPX, and CAT in the colonic mucosa, which promotes spontaneous obesity [138]. Additionally, AST-fed db/db mice showed decreased colonic NF-κB+ and PCNA+ cell counts as well as decreased gene expressions of IL-1β, IL-6, F4/80, chemokine (C-C motif) ligand 2 (CCL2), and chemokine (C-X-C motif) ligand 2 (CXCL2). In general, these findings point to AST’s anti-oxidant, anti-inflammatory, and anti-metastasis properties, which may make it an interesting option for a disease chemoprevention medication.

### 6.3. Gastric Ulcer

The characteristic feature of a gastric ulcer is a deep necrotic lesion in the gastroduodenal mucosa that breaks the normal integrity of the gastric mucosa and extends into the submucosa through the muscularis mucosa [182]. Considerable histologic and ultrastructural abnormalities are involved, including decreased height, the gastric glands’ considerable dilatation, the glandular cells’ poor differentiation and/or degenerative alterations, the enlarged connective tissue, and the disturbed microvascular network [183]. Various stimuli, including cigarette smoking, alcohol use, bacterial infections, UV exposure, and NSAID administration, cause the stomach to create ROS [184]. Among these, oxidative stress caused by an *H. pylori* infection or by NSAID consumption such as ibuprofen and aspirin in humans is the primary cause of stomach ulcers. Reactive nitrogen species (RNS) and ROS are produced when neutrophils migrate to the inflammatory area in response to an *H. pylori* infection or the consumption of NSAIDs. Nicotinamide adenine dinucleotide phosphate (NADPH) oxidase and mucosal xanthine oxidase, which are found in resident leukocytes and gastric epithelial cells, are among the sources of radicals [185]. Increased ROS upregulate inflammatory genes by trigger the activation of redox-sensitive transcription factors NF-κB and AP-1, and in turn, enhance the expression of adhesion molecules and inflammatory cytokines like IL-8 [186]. Thus, oxidative stress is linked to the degree of inflammation in the stomach mucosa.

Rats treated with naproxen showed protection against naproxen-induced stomach ulcers when given AST (1, 5, or 25 mg/kg BW) twice daily for three days. This was achieved by correcting reduced SOD, CAT, and GPX activities and raising the lipid peroxide level to that of untreated normal rats [187]. In response to the ethanol-induced gastric ulcer in rats, AST pretreatment (5 or 25 mg/kg BW; three days) significantly improved the activity of antioxidant enzymes such as GPX, SOD, and CAT [188]. Furthermore, AST may control the secretion of mucin and stomach acid. In rats administered ethanol, AST pretreatment (100, 250, or 500 µg/kg BW; three weeks) increased mucin content and inhibited the gastric H+, K+-ATPase proton pump, protecting the gastric mucosal layer from oxidative stress and excessive gastric acid secretion in the stomach cells [189]. Similarly, in young adult ddY mice, pretreatment with AST (30 or 100 mg/kg BW; 1 h) before ethanol/hydrochloride administration reduced the total lesion area and prevented disruption of the superficial regions of the gastric gland with loss of epithelial cells. It also decreased lipid peroxidation and histological damage in the superficial layers of the gastric mucosa. Such findings were reported by [190]. The effects of AST have also been investigated using a model of stress-induced stomach ulcers. In this model, rats were submerged in chest-level water for 24 h to create stress [191]. In response to water-induced stress, rats given AST (40 mg/100 g food) beforehand showed significantly lower ulcer indices and stress-related stomach damage than rats given control.

A Gram-negative bacterium *H. pylori* is strongly linked to the emergence of gastrointestinal disorders such gastritis, peptic ulcers, and gastric cancer [192]. Increased risk for atrophic gastritis, gastric ulcers, and the pathophysiology of gastric cancer is associated with the cytotoxin-associated gene A (CagA) and the generation of a vacuolating cytotoxin encoded by the vacuolating cytotoxin A (VacA). *H. pylori* can damage the protective lining of the stomach and small intestine, allowing the acid to induce an open sore that leads to gastric ulcer and duodenal ulcer, respectively [193]. It has been demonstrated that AST protects gastric epithelial cells against *H. pylori* infection by reducing cytokine and ROS production. Peroxisome proliferator-activated receptor-γ (PPAR-γ) was activated and its downstream antioxidant gene, such as catalase, was enhanced in *H. pylori*-infected gastric epithelial cells by AST treatment (1 or 5 µM; 3 h), according to Kim et al. Thus, AST reduced intracellular and mitochondrial ROS formation, as well as ROS-mediated NF-κB activation and IL-8 expression [129]. In terms of mechanism, the anti-oxidant qualities of AST in gastric epithelial cells prevented adenosine triphosphate (ATP) depletion and mitochondrial dysfunction brought on by *H. pylori*. Additionally, the research team showed that AST (1 or 5 µM; 3 h) prevented *H. pylori*-induced decreases in superoxide dismutase 2 (SOD2) level and SOD activity and reduced mitochondrial ROS in gastric epithelial cells [194]. Numerous in vivo investigations have been conducted with mouse models infected with *H. pylori*. For C57BL/6 mice inoculated with *H. pylori*, feeding them a standard chow diet supplemented with AST (5 mg/kg BW; 7 weeks) significantly decreased oxidative damage to gastric mucosa cells by reducing the increase in lipid peroxide (LPO) production, myeloperoxidase (MPO) activity, expression of the inflammatory cytokine IFN-γ, and oncogenes such as c-myc and cyclin D9 [195]. Bennedsen et al. found that oral supplementation of AST-rich micro algae, *Haematococcus pluvialis*, at a dose of 200 mg/kg BW for 10 days significantly reduced gastric inflammation and bacterial load in BALB/c mice infected with *H. pylori* through modifying the T-lymphocyte response from a Th1-dominant to a Th1/Th2-balanced state [128]. Another study provided additional evidence for this concept. Oral AST delivery (10 or 40 mg/kg BW; 6 weeks) enhanced the release of Th2-type cytokines like IL-2 and IL-10 in BALB/c female mice infected with *H. pylori*, causing a shift in splenocytes toward a balanced Th1/Th2 response [127]. In a randomised double-blind, placebo-controlled clinical investigation, 42 young, healthy females (average age: 21.5 years) were given dietary AST (2 or 8 mg/kg BW) for eight weeks. The AST capsule contained oleoresin concentrate from *Haematococcus puvialis*, and the participants showed decreased levels of plasma acute phase protein (C-reactive protein) and oxidative DNA damage biomarker (8-hydroxy-2′-deoxyguanosine) because it stimulated mitogen-induced lymphoproliferation and increased natural killer cell cytotoxic activity and total T and B cell subpopulations [39]. Andersen et al. conducted a randomized clinical experiment in which they gave AST capsules (20 mg AST twice day for eight weeks) or placebo capsules (23 patients, dextrin-filled capsule) to 44 patients (average age: 51 years) who had both functional dyspepsia and an *H. pylori* infection. *Haematococcus puvialis* algae meal was present in the active AST capsule [99]. Patients treated with AST demonstrated a considerable up-regulation of T helper cells (CD4) and a down-regulation of cytotoxic T cells (CD8), which led to a decrease in gastric inflammation even though there were no changes in the *H. pylori* density. The local ethics committee accepted this study, which complied with the second Helsinki Declaration [130]. All of these findings indicate the potential use of AST as a treatment for ulcers and damage to the stomach mucosa via regulating oxidative stress and the immune system.

### 6.4. Inflammatory Bowel Diseases (IBD)

The term “inflammatory bowel diseases” (IBDs) refers to idiopathic, chronic, recurrent gastrointestinal inflammatory disorders. Due to its varying geographic increases in incidence and prevalence, IBD is becoming a more prevalent disease. A growing body of research indicates that the intricate interaction of genetic, immunologic, and environmental variables that can be changed causes IBDs [196]. Ulcerative Colitis (UC), a chronic inflammatory bowel disease, is becoming more commonplace worldwide [197]. Patients with ulcerative colitis frequently have diarrhea and blood in their stool, and the condition is defined by persistent mucosal inflammation that spreads from the rectum to the proximal colon [198]. Ulcerative colitis has a complex etiology that involves environmental factors like nutrition, immune system abnormalities, gut barrier deficiencies, and genetic predisposition [199]. The main characteristics of ulcerative colitis include increased intestinal permeability in intestinal mucosa and epithelial crypts, pro-inflammatory transcription factors and cytokines being activated, and increased oxidative stress caused on by neutrophil accumulation [200].

In a mouse model of DSS-induced colitis, the dietary pretreatment of AST (0.02–0.04% mixed in a rodent chow diet; seven days) before to the administration of DSS suppressed the mucosal gene expression of pro-inflammatory cytokines such as IL-1β, IL-6, TNF-α, and IL-36 and significantly decreased the body weight loss brought on by DSS [140]. Yasui et al. [139] also reported similar results, in mice with DSS-induced colitis, mice fed experimental diets containing AST (100–200 ppm) showed suppressed NF-κB expression in the colon and decreased gene expressions of several inflammatory markers, including TNF-α, IL-1β, IL-6, COX-2, and iNOS. Zhang et al. conducted another investigation in which they used cauliflower-like carriers to create AST-loaded nanocarriers and evaluated the particles in vitro and in mice given DSS. According to in vitro research, AST-loaded nanocarriers significantly enhanced AST internalization and declined ROS generation in the mitochondria by more efficiently targeting the mitochondria than a free form of AST. Furthermore, in vivo experiments with BALB/c mice treated with DSS demonstrated that AST-loaded nanocarriers (250 mg/kg; 13 days) conserved the integrity of the colon tissue by suppressing the expression of IL-1β, IL-6, COX-2, MPO, and iNOS and by promoting the expression of the intestinal tight junction protein zonula occludens-1 (ZO-1) [201]. In a rat model of experimental necrotizing enterocolitis, AST (100 mg/kg BW; four days; oral gavage) significantly decreased intestinal damage because it decreased oxidative stress (higher levels of GSH and SOD), inflammation (lower levels of IL-1β and TNF-α), and apoptosis (lower levels of caspase-3). Rats given AST then responded to necrotizing enterocolitis with higher weight growth and survival rates [202]. The addition of AST-enriched yeast (120 mg/kg BW; 7–21 days) also increased immunoglobulin A (IgA) levels in the ileum and jejunum of weanling mice [203]. These results in rodent models highlight the prospect of nutraceutical potential in ulcerative colitis by indicating that AST suppresses the disease by reducing oxidative stress and inflammation. Nevertheless, no clinical trials have been performed.

### 6.5. Gut Microbiota

The gut microbiota, a vital component of the human body, is an ecosystem with about 5000 species that affects a wide range of biological and metabolic processes factors including the host gene, diet, lifestyle, and medications can all affect it. Unbalanced gut microbiome homeostasis, or dysbiosis, may be a contributing factor to inflammation and metabolic conditions like cancer, diabetes, and obesity [204,205,206].

Recent studies have documented the beneficial effects of AST on gut microbiota under various situations. For instance, study by Liu et al. [207] investigated how AST affected the gut microbiota’s composition, ethanol-induced liver lesions, and the alcoholic fatty liver disease phenotype in a model of mice fed ethanol. A high-fat diet fed to mice, the study examined the effects of (3R,3′R)-AST from X. dendrorhous on gut microbiota and lipid metabolism. AST increased the amount of *Verrucomicrobia*, particularly *Akkermansia*, and optimized the ratio of *Bacteroides* to *Firmicutes*. In 8 groups of mice, 4 of them were given either AST or X. dendrorhous food (AST 0.005%, AST 0.01%, X. dendrorhous 10% *w*/*w*, and X. dendrorhous 20% *w*/*w*, respectively) for 8 weeks. *Akkermansia* is a probiotic, and the ratio of *Bacteroides* to *Firmicutes* influences how well the body absorbs calories from food, which is a major factor in changes in body weight [208]. Similarly, it was demonstrated that AST (50 mg/kg body weight) effectively reversed the negative effects of a high-fat plus-ethanol diet on the composition and structure of gut microbiota after 12 weeks [209]. In a recent study, AST treatment dramatically increased the population of *Mucispirillum schaedleri* and *Akkermansia muciniphila* compared to wild type mice using a model of β-carotene oxygenase knockout mice (C57BL/6 mice). However, it was shown that male and female mice have different gut microbiome topologies [210]. Likewise, in a different pilot study, giving AST to β-carotene oxygenase knockout mice’s diet altered the gut microbial genera; this modification was different for both genders of mice and was similarly closely linked to β-carotene oxygenase gene expression. In addition, it was discovered that wild-type mice have higher levels of commensal microbiota, including *Actinobacteria* and *Bifidobacterium* [211]. Consequently, AST therapy may significantly alter the composition of the gut microbiota, reducing the incidence of gut disorders. In addition to its advantageous benefits on gut health, it is important to examine AST’s influence on skin health. The correlation between internal health and outward beauty highlights the significance of nutrients such as AST, which can enhance digestive function while fostering healthier, more youthful skin.

## 7. Astaxanthin and Skin Health

Many components, such as cornified envelopes, corneocytes, lipids, junctional proteins, proteases, protease inhibitors, antimicrobial peptides, and transcription factors, have been linked to the development of epidermal barriers. The epidermis, dermis, and hypodermis create the skin’s protective outermost barrier against external environmental stresses like repeated exposure to ultraviolet (UV) rays, invasion by microbes or pathogens, physicochemical agents, and excessive transpiration of internal fluids during the day [212]. Conversely, long-term exposure to the UV rays of the sun causes skin photoaging, which is clinically defined by deep wrinkles, pigmentation, dryness, and laxity [213]. The main cause of skin cancer in both people and animals is exposure to UVB radiation, which also causes reactive oxygen species to develop and adversely destroys cellular macromolecules [214]. UVB primarily damages the epidermis and DNA, whereas UVA can reach the dermis and harm dermal collagen and elastin [215]. Skin changes include pigment buildup, weakening skin layers, and a lack of suppleness and oil gland function. Wrinkles, age spots, and dry, loose, or sagging skin are caused by these and other factors [3]. Therefore, reducing the consequences of skin damages through various therapeutic is vital to ensuring a high degree of skin well-being.

AST has demonstrated anti-inflammatory, immune-modulating, and DNA-repairing properties that can effectively preserve skin health, encouraging normal, healthy skin by increasing skin moisture and suppleness and reducing wrinkle formation [216]. It has been shown that AST supplementation (4 mg/day) for four weeks improves skin in people over 40 by lowering corneocyte desquamation and lipid oxidation. AST’s positive outcomes were primarily due to its antioxidant qualities [217]. A study by Tominaga et al. was conducted out in Japan in 2017 to assess the anti-inflammatory effects of AST on skin degeneration. An initial in vitro study revealed that AST therapy reduced the production of matrix metalloproteinase-1 (MMP-1) by fibroblasts cultured in media of UV-B-induced keratinocytes and controlled the inflammatory response related to UV-B in the keratinocytes. After then, 65 healthy women were enrolled in a 16-week clinical research. AST (6 mg or 12 mg) or a placebo was given to the participants. It has been found that long-term AST supplementation can assist reduce aging-induced skin deterioration and maintain skin health based on evaluation of wrinkle parameters or skin moisture levels [218]. Another study demonstrated that by reducing the expression of aquaporin 3 and other proteins, AST decreased the transepidermal water loss linked to UV exposure, consequently minimizing skin damage [219]. Study by Chung et al. discovered experimentally that AST expressively inhibited the ultravioletinduced cytotoxicity and cell death of epidermal keratinocytes [220]. When AST was given, the skin’s collagen fibers grew and UV-induced wrinkles decreased [221]. Furthermore, the advantages of AST supplementation (3–6 mg/d) for photoaged skin are supported by clinical trials [218,222].

Based on studies on humans, taking 6 mg of AST daily for 6 to 8 weeks may help prevent wrinkles, hydration loss, and age spots. In addition, AST improved skin texture, elasticity, and moisture content; these benefits appear to be enhanced when applied topically [159,223]. A recently published Japanese study used a double-blind, randomized, placebo-controlled design to assess AST preventive effect against UV-induced skin degeneration in healthy individuals. Study in 2018, a 23 healthy participants were given either a placebo or a 4 mg AST capsule by Ito et al. Along with a subjective visual analogue scale for the individuals, the minimum erythema dose (MED) and transepidermal water loss (TEWL) were determined at baseline and nine weeks following AST admin. When compared to the placebo group, it was discovered that the AST group had higher MED and decreased skin moisture loss from the irradiation areas. Subjective characteristics of the skin, like smoothness and roughness, improved. According to the researchers’ findings, AST helps maintain healthy skin and protects against UV-induced skin damage [224]. In comparison to the placebo group, AST supplementation intensely decreased skin damage and assisted in preserving skin hydration [223]. The key conclusions from the human research that examined AST positive dermatological effects are summarized in Table 4.

## 8. Astaxanthin Toxicity

While usually regarded safe, various issues with high-dose exposure and specific populations have been investigated. A rat carcinogenicity research using dietary doses of 40, 200, and 1000 mg AST/kg bw/day revealed an increased incidence of benign hepatocellular adenoma in female rats at 200 mg/kg bw/day and higher. However, there was no apparent dose–response connection for this observation, and genotoxicity experiments, including Ames and in vitro Micronucleus Tests, revealed no genotoxic potential for synthetic AST. This indicates that AST is unlikely to induce genetic harm. The presence of hepatotoxicity, as evidenced by liver adenomas in rats, emphasises the significance of dose-dependent effects and species-specific responses in toxicology. In a one-year chronic trial in rats, symptoms of mild toxicity were detected in the form of alterations in clinical chemistry, such as increased blood cholesterol and bilirubin at daily dosages of 125 mg/kg bw/day or higher. Furthermore, in life-long trials in rats and mice, a beadlet formulation with 8% AST was not carcinogenic [229,230]. Another study found that rats’ dietary exposure to AST-rich microalgae biomass for 90 days had no biologically significant negative effect on a number of health-related measures [231]. Another study that treated rats with synthetic (3S, 3′S)-AST for 13 weeks found similar results [232]. Both investigations indicated a colour change in faeces or GI tract content that was linked to AST colouration impact. Continuous monitoring and strict use of toxicological principles, including the determination of NOAELs and safety margins, are crucial for assuring the safe use of AST, especially as new sources and formulations arise.

## 9. Future Perspectives

Although substantial progress has been made, research on the anti-inflammatory effects of AST in bacterial infections remains limited. Similarly, data concerning its potential impact on viral and fungal infections are scarce, in contrast to the extensive body of evidence available for other chronic and acute conditions [233], There is a clear need for well-designed, large-scale, long-term clinical trials in humans. Many existing studies are constrained by small sample sizes, brief intervention periods, and heterogeneous participant populations. To fully elucidate AST’s mechanisms and long-term health effects across diverse populations and clinical contexts, more rigorous and comprehensive trials are essential.

As research advances, opportunities will emerge to develop personalized nutrition strategies that tailor AST supplementation to individual health needs, particularly among older adults. Innovations in delivery systems—such as liposomal formulations and microencapsulation—hold promise for enhancing AST’s bioavailability, tissue targeting, and overall therapeutic efficacy. Future investigations should also explore its effects on a wider range of conditions, including metabolic disorders, neurological diseases, fungal infections, and specific cancers. Finally, translational studies examining synergistic combinations of AST with other nutraceuticals or pharmacological agents could yield additive or synergistic benefits, especially in complex pathologies such as metabolic syndrome, neurodegenerative diseases, and immunosenescence. Through these avenues, AST has the potential to evolve from a promising nutraceutical into a well-established therapeutic component of precision nutrition and integrative medicine.

## 10. Conclusions

We conclude that AST emerges as a potent and versatile bioactive compound with significant promise for promoting human health, especially aging, oxidative stress, immune dysfunction, gut health, and skin vitality. Its strong antioxidant qualities aid in reducing oxidative damage, a major cause of aging and a number of chronic diseases. Furthermore, its immunomodulatory properties suggest a role in restoring immune homeostasis, thereby enhancing resilience against both inflammatory pathologies and infectious challenges. The emerging evidence regarding gut microbiota modulation and dermal protection further expands the therapeutic landscape of AST, positioning it not merely as an antioxidant but as a pleiotropic functional compound capable of influencing the gut–skin axis and systemic health. Furthermore, AST ability to enhance skin elasticity and hydration as well as its protection against UV-induced skin damage highlight its significance in dermatological applications. Additional investigation into the mechanisms of action and long-term effects of AST is necessary in light of the rising incidence of age-related disease and the growing interest in preventative health condition. Collectively, these findings advocate for AST as a promising nutritional strategy for promoting healthy aging and managing chronic degenerative conditions.

## Figures and Tables

**Table 2:** Effects of AST on aging-related diseases.

Subjects	Dosage mg/Day	Treatment Duration	Outcomes	References
27 patients with non-advanced AMD	4	12 months	Increase multifocal electroretinogram RAD for retinal eccentricity of 0° to 5°	[24]
26 VDT workers	5	4 weeks	Improvement of accommodation amplitude	[72]
24 healthy volunteers	1.8, 3.6, 14.4, 21.6	14 days	Increase LDL oxidation lag time	[73]
42 healthy young women subjects	2, 8	8 weeks	Decrease CRP and 8-OHdG, Increase NK cell cytotoxic activity, Increase IFN-γ and IL-6, Increase total T and B cells	[39]
40 healthy men volunteers	8	3 months	Decrease the levels of plasma 12- and 15-hydroxy fatty acid	[23]
Age-related forgetfulness	6, 12	12 weeks	Increase scores in the GMLT cognitive tests and Cog Health	[74]
10 subjects with age-related forgetfulness	12	12 weeks	Increase performances in Cog Health and P300 cognitive tests	[21]
39 heavy smoker participants	5, 20, 40	3 weeks	Decrease ISP and MDA, Increase TAC and SOD plasma levels	[75]
23 overweight and obese healthy adults	5, 20	3 weeks	Decrease MDA and ISP, Increase SOD and TAC plasma level	[26]
30 middle-aged and senior healthy subjects	6, 12	12 weeks	Decrease erythrocyte PLOOH levels	[76]
61 healthy participants with mild hyperlipidemia	6, 12, 18	12 weeks	Decrease triglyceride, Increase HDL-cholesterol and adiponectin	[77]
12 biopsy-confirmed NASH patients	12	24 weeks	Decrease total NAS score, improvement of steatohepatitis,	[78]
20 healthy adult men participants	6	10 days	Decrease whole blood transit time (Unit: Sec/100 µL)	[79]

Abbreviations: AMD, age-related macular degeneration; RAD, response amplitude densities; VDT, visual display terminal; LDL, low-density lipoprotein; CRP, C-Reactive protein; 8-OHdG, 8-hydroxy-2’-deoxyguanosine; NK, natural killer; IFN, interferon; IL, interleukin; GMLT, Groton maze learning test; ISP, isoprostane; MDA, malondialdehyde; TAC, total antioxidant capacity; SOD, superoxide dismutase; PLOOH, phospholipid hydroperoxide; HDL, high-density lipopro-tein; NASH, non-alcoholic steatohepatitis; NAS, NAFLD activity score.

**Table 3:** Effects of AST on inflammatory pathways.

Condition	Subject	Dosage	Outcomes	Reference
*H. pylori* infection	Female BALB/c mice	100 mg/kg	Inhibit *H. pylori* Infection and reducing IFN-γ, IL-4, and bacterial load	[126]
*H. pylori* infection	BALB/c female mice	10 or 40 mg/d	Inhibit *H. pylori* Infection and restored IFN-γ, IL-2 and IL-10 concentrations	[127]
*H. pylori* infection	Balb/cA mice	200 mg per kg body weight per day	Inhibit *H. pylori* Infection and reduced IFN-γ, IL-2, IL-4, and bacterial load	[128]
*H. pylori* infection	Human gastric epithelial cell line AGS	5 µM	Inhibit *H. pylori* Infection and restored ROS, PPAR-γ, NF-κB, and IL-8 concentrations	[129]
*H. pylori* infection	Patients	40 mg daily	Inhibit *H. pylori* Infection and restored CD4, and CD8	[130]
ConA-induced autoimmune hepatitis	Male Balb/c mice	20 mg/kg and 40 mg/kg	Hepatoprotective effect by restoring NF-κB p65, TNF-α, IL-6, IL-1β, IFN-γ, apoptotic proteins and autophagy	[131]
Hepatic ischemia–reperfusion (IR)	Male C57BL/6 mice	25 mg/kg	Hepatoprotective effect by restoring ROS, inflammatory cytokines, MAPK and apoptosis-related proteins	[132]
Hepatic ischemia–reperfusion (IR)	Male Balb/C mice	30 mg/kg or 60 mg/kg	Hepatoprotective effect by restoring ROS, inflammatory cytokines and MAPK proteins	[133]
Ischemia/reperfusion (IR) induced injury	Male ICR mice	5 mg/kg/day	Nephroprotective effect by restoring TNF-α, IL-1β, and IL-6	[134]
Adriamycin-induced FSGS	Male Balb/c mice	50 mg/kg	Nephroprotective effect by restoring Nrf2, NLRP3, IL-1β, and IL-18	[135]
Contrast-induced acute kidney injury (CI-AKI)	Male Sprague Dawley (SD) rats	5 mg/kg/day	Nephroprotective effect by restoring oxidative stress indicators, and antioxidant stress indicators	[136]
Contrast-induced acute kidney injury (CI-AKI)	Male Sprague Dawley rats	50 and 100 mg/kg	Nephroprotective effect by restoring oxidative stress markers and apoptosis-related proteins	[137]
Azoxymethane-induced colonic premalignant lesions	C57BL/KsJ-db/db obese mice	200 ppm in diet	Gastroprotective effect by restoring NF-κB, IL-1β, IL-6, CCL2, and CXCL2	[138]
Dextran sulfate sodium (DSS)-induced colitis	Male ICR mice	50, 100, 200 ppm in diet	Gastroprotective effect by restoring NF-κB, IL-1β, IL-6, and COX-2	[139]
Colitis induced by Dextran Sulfate Sodium (DSS)	C57BL/6J mice	0.02 or 0.04% in diet	Gastroprotective effect by restoring TNF-α, IL-1β, IL-6, IL-36α, IL-36γ, NF-κB, AP-1, ERK1/2, p38 MAPK, and JNK	[140]
Neuropathic pain	Rat C6 glial cells; Adult male Sprague Dawley rats	5 and 10 mg/kg	Neuroprotective effect by restoring ROS	[141]
Neuropathic pain	Spinal cord injury (SCI) rats	10 µL of 0.2 mM	Neuroprotective effect by restoring NR2B, p-p38MAPK and TNF-α	[142]
Neuropathic pain	Chronic constriction injury (CCI) mice	80 mg/kg	Neuroprotective effect by restoring IL-1β, IL-6 and TNF-α	[143]
Neuropathic pain	Adult male Wistar rats	10 µL of 0.2 mM	Neuroprotective effect by restoring p-p38MAPK, NR2B, and TNF-α	[144]
Edema and pain	Male ICR mice	50, 100, 150 mg/kg	Neuroprotective effect by restoring ROS	[145]
Cerebral ischemia	Human SH-SY5Y cells	5, 10, 20 and 40 µmol/L	Neuroprotective effect by restoring GSK3β/PI3K/Akt/Nrf2 signalling	[146]
Acute cerebral infarction	Male Sprague Dawley rats	20, 40, and 80 mg/kg	Neuroprotective effect by restoring oxidative stress	[147]
Cerebral ischemia	Adult male Sprague-Dawley rats	20, 40, and 80 mg/kg	Neuroprotective effect by restoring oxidant parameter	[148]
Cerebral ischemia	MCAO mice	30 mg/kg	Neuroprotective effect by restoring cAMP concentration	[149]
Alzheimer’s Disease	Wistar rats	10 mg/kg body weight	Neuroprotective effect by restoring oxidative markers	[150]
Cerebral ischemia/ reperfusion (IR)	Male ICR mice	10 mg/kg/day	Neuroprotective effect by restoring Parameters of oxidative stress: cleaved Caspase-3, Bax, and Cytochrome C	[151]
Cerebral ischemia	Male SD (Sprague-Dawley) rats	10 mg/kg or 5 mg/kg	Neuroprotective effect by restoring genes for oxidative stress, antioxidants, cell death detection, and cell regeneration	[152]
Alzheimer’s Disease	APP/PS1 mice	10 mg/kg body weight	Neuroprotective effect by restoring oxidative markers; inflammasome expression	[153]
Parkinson’s disease	Mice	Bioastin^®^ at a dose of 30 mg/kg bodyweight	Neuroprotective effect by restoring MPTP neurotoxin	[154]
Parkinson’s disease	Human neuroblastoma SH-SY5Y cell line and C57BL/6 mice	5, 10, 25, and 50 µM in cell line	Neuroprotective effect by restoring miR-7/SNCA axis	[155]
Parkinson’s disease	Mice with Parkinson’s disease (PD),	5, 10, 25, and 50 µM in cell line	Neuroprotective effect by restoring the JNK and P38 MAPK pathways are examples of the mitochondria-mediated route.	[156]
Dry eye disease	BALB/c mice	1 µL drop of 5 µM	Eye-protective effect by restoring PI3K/Akt, HMGB1, TNF-α, and IL-1β	[157]
Dry eye disease	Male SpragueDawley rats	200 µM	Eye-protective effect by restoring DED-related factors	[158]
Atopic dermatitis	HR-1 mice	10 µg or 20 µg/cm^2^	Skin-protective effect by restoring TNF-α, IgE, COX-2, NF-κB, iNOS, IL-1β, and IL-6	[159]
Atopic dermatitis	Male NC/Nga mice	100 mg/kg	Skin-protective effect by restoring MIF, eotaxin, L-histidine decarboxylase, IL-4, and IL-5	[160]

**Table 4:** Summary of the major findings on the positive dermatological effects of AST in skin-related conditions.

Subject	Concentration/Dosage per Day	Administration	Outcome	Reference
Human	4 mg	Oral	A strong antioxidant effect and facial skin rejuvenation	[225]
Human	4 mg	Oral	Reduced skin moisture loss, increased minimal erythema dose (MED), and improved skin texture	[224]
Mice	20 J/cm^2^	Topical	Preventing UV radiation-induced photoaging	[219,221]
Human	6 mg or 12 mg	Oral	Improvement of skin conditions and prevention of age-related skin damage	[218]
Mice	78.9 µM	Topical	Wound showed noticeable contraction by day 3 of treatment and complete wound closure by day 9	[226]
Human	3 mg, 1 mL	Oral, Topical	Reduced skin wrinkles and age spots, enhanced skin’s moisture content, texture, and elasticity decrease in TEWL and crow’s feet wrinkles	[227]
Human	4 mg	Oral	Reduced skin microbial activity, reduced desquamation of corneocytes, and antioxidant activity that results in facial renewal	[217]
Human	2 mg, collagen hydrolysate 3 g	Oral	improved skin elasticity and a decrease in TEWL from photoaged face skin	[228]

## Data Availability

No new data were created or analyzed in this study. Data sharing is not applicable to this article.

## Supplementary Materials

The following supporting information can be downloaded at: https://www.mdpi.com/article/10.3390/antiox15050575/s1, Table S1: Shows the differences between natural and synthetic astaxanthin (AST) in encompassing efficacy, safety, and market preference.

## Abbreviations

The following abbreviations are used in this manuscript:

ATP	Adenosine Triphosphate
AST	Astaxanthin
BDNF	Brain-Derived Neurotrophic Factor
BrdU	Bromodeoxyuridine
CagA	Cytotoxin-associated gene A
CCL2	Chemokine (C-C motif) Ligand 2
COX-2	Cyclooxygenase-2
CRP	C-reactive protein
CXCL2	Chemokine (C-X-C motif) Ligand 2
DNA	Deoxyribonucleic Acid
GSH	Glutathione
GPX	Glutathione Peroxidase
GST-1	Glutathione S-transferase 1
HO-1	Hemeoxygenase 1
iNOS	Inducible Nitric Oxide Synthase
IgA	Immunoglobulin A
KLF4	Kruppel-like Factor 4
MMP-1	Matrix Metalloproteinase-1
MED	Minimum Erythema Dose
NF-κB	Nuclear Factor kappa-light-chain-enhancer of activated B cells
NPCs	Neural Progenitor Cells
Oct4	Octamer-binding Transcription Factor 4
PI3K	Phosphoinositide 3-Kinase
PPARγ	Peroxisome proliferator-activated receptor gamma
ROS	Reactive Oxygen Species
RONS	Reactive Oxygen and Nitrogen Species
SOD1	Superoxide Dismutase 1
TEWL	Transepidermal Water Loss
TNF-α	Tumor Necrosis Factor-alpha
VacA	Vacuolating Cytotoxin A
ZO-1	Zonula Occludens-1
