# Supplementary material for: Exploring the Multifunctional Benefits of Astaxanthin in Aging, Oxidative Stress, Immune Dysfunction, Gut and Skin Health
Source: Antioxidants (Basel). 2026 May 2;15(5):575. doi: 10.3390/antiox15050575 (PMC13203560; doi:10.3390/antiox15050575)
Supplement: Supplementary file 1 [file antioxidants-15-00575-s001.zip › antioxidants-4172443-supplementary.pdf]

Table S1: Shows the differences between natural and synthetic astaxanthin (AST) in encompassing efficacy, safety, and market preference

	**Natural Astaxanthin**	**Synthetic Astaxanthin**
**Source**	Predominantly derived from microalgae, specifically *Haematococcus pluvialis*, as well as from yeast and several marine species [1-4].	Chemically synthesized [4-6]
**Structure**	Predominantly 3S,3'S stereoisomer, often esterified [3]. This stereoisomeric configuration is thought to enhance its biological efficacy and antioxidant characteristics [7].	Typically a racemic mixture of several stereoisomers (e.g., 3S, 3′ S, 3R, 3′ R, and meso forms) [3].
**Bioactivity**	Recognized for its powerful antioxidant, anti-inflammatory, and many medicinal properties [1,2,7]. It has demonstrated superior efficacy in mitigating oxidative and free radical stress compared to synthetic AST [1]. Natural AST sourced from *Haematococcus pluvialis* can mitigate lipid peroxidation and ferroptotic cell death [7].	While effective, certain studies suggest it may have less significant biological effects than natural AST [1]. However, in aquaculture, synthetic AST has shown equivalent effects to natural sources in increasing growth, pigmentation, and antioxidant capacity in several species [8,9].
**Applications**	Preferred for human nutraceuticals, cosmetics, and pharmaceuticals owing to its perceived natural origin and augmented bioactivity [10-12]. Its production is considered eco-friendly.	It dominates the aquaculture feed market because of its low cost and effectiveness in coloration and growth enhancement in aquatic animals [5,13].
**Cost**	Generally costlier to produce than synthetic counterparts owing to intricate cultivation and extraction methods [5,6].	Cheaper to produce, making it economically appealing for large-scale commercial applications, particularly in animal feed [5,6].

References

1. Kumar, S.; Kumar, R.; undefined, u.; Kumari, A.; Panwar, A. Astaxanthin: A super antioxidant from microalgae and its therapeutic potential. *Journal of Basic Microbiology* **2021**, *62*, 1064-1082, doi:10.1002/jobm.202100391.
2. Aneesh, P.A.; Ajeeshkumar, K.K.; Lekshmi, R.G.K.; Anandan, R.; Ravishankar, C.N.; Mathew, S. Bioactivities of astaxanthin from natural sources, augmenting its biomedical potential: A review. *Trends in Food Science & Technology* **2022**, *125*, 81-90, doi:10.1016/j.tifs.2022.05.004.
3. Nair, A.; Ahirwar, A.; Singh, S.; Lodhi, R.; Lodhi, A.; Rai, A.; Jadhav, D.A.; Harish, u.; Varjani, S.; Singh, G.; et al. Astaxanthin as a King of Ketocarotenoids: Structure, Synthesis, Accumulation, Bioavailability and Antioxidant Properties. *Marine Drugs* **2023**, *21*, 176, doi:10.3390/md21030176.
4. Villaró, S.; Ciardi, M.; Morillas-España, A.; Sánchez-Zurano, A.; Acién-Fernández, G.; Lafarga, T. Microalgae Derived Astaxanthin: Research and Consumer Trends and Industrial Use as Food. *Foods* **2021**, *10*, 2303, doi:10.3390/foods10102303.

5. Debnath, T.; Bandyopadhyay, T.K.; Vanitha, K.; Bobby, M.N.; Nath Tiwari, O.; Bhunia, B.; Muthuraj, M. Astaxanthin from microalgae: A review on structure, biosynthesis, production strategies and application. *Food Research International* **2024**, *176*, 113841, doi:10.1016/j.foodres.2023.113841.
6. Acheampong, A.; Li, L.; Elsherbiny, S.M.; Wu, Y.; Swallah, M.S.; Bondzie-Quaye, P.; Huang, Q. A crosswalk on the genetic and conventional strategies for enhancing astaxanthin production in <i>Haematococcus pluvialis</i>. *Critical Reviews in Biotechnology* **2023**, *44*, 1018-1039, doi:10.1080/07388551.2023.2240009.
7. Rizzardi, N.; Pezzolesi, L.; Samorì, C.; Senese, F.; Zalambani, C.; Pitacco, W.; Calonghi, N.; Bergamini, C.; Prata, C.; Fato, R. Natural Astaxanthin Is a Green Antioxidant Able to Counteract Lipid Peroxidation and Ferroptotic Cell Death. *International Journal of Molecular Sciences* **2022**, *23*, 15137, doi:10.3390/ijms232315137.
8. Huang, S.; Chen, Q.; Zhang, M.; Chen, S.; Dai, J.; Qian, Y.; Gong, Y.; Han, T. Synthetic astaxanthin has better effects than natural astaxanthins on growth performance, body color and n-3 PUFA deposition in black tiger prawn (Penaeus monodon). *Aquaculture Reports* **2023**, *33*, 101816, doi:10.1016/j.aqrep.2023.101816.
9. Wang, L.; Long, X.; Li, Y.; Zhang, Y.; Sun, W.; Wu, X. Effects of Three Sources of Astaxanthin on the Growth, Coloration, and Antioxidant Capacity of Rainbow Trout (Oncorhynchus mykiss) during Long-Term Feeding. *Fishes* **2024**, *9*, 174, doi:10.3390/fishes9050174.
10. Rodríguez-Sifuentes, L.; Marszalek, J.E.; Hernández-Carbajal, G.; Chuck-Hernández, C. Importance of Downstream Processing of Natural Astaxanthin for Pharmaceutical Application. *Frontiers in Chemical Engineering* **2021**, *2*, doi:10.3389/fceng.2020.601483.
11. Patel, A.K.; Tambat, V.S.; Chen, C.-W.; Chauhan, A.S.; Kumar, P.; Vadrale, A.P.; Huang, C.-Y.; Dong, C.-D.; Singhania, R.R. Recent advancements in astaxanthin production from microalgae: A review. *Bioresource Technology* **2022**, *364*, 128030, doi:10.1016/j.biortech.2022.128030.
12. Cao, Y.; Yang, L.; Qiao, X.; Xue, C.; Xu, J. Dietary astaxanthin: an excellent carotenoid with multiple health benefits. *Critical reviews in food science and nutrition* **2021**, *63*, 3019-3045, doi:10.1080/10408398.2021.1983766.
13. Elbahnaswy, S.; Elshopakey, G.E. Recent progress in practical applications of a potential carotenoid astaxanthin in aquaculture industry: a review. *Fish Physiology and Biochemistry* **2023**, *50*, 97-126, doi:10.1007/s10695-022-01167-0.
